# Systematic profiling of ACE2 expression in diverse physiological and pathological conditions for COVID‐19/SARS‐CoV‐2

**DOI:** 10.1111/jcmm.15607

**Published:** 2020-07-08

**Authors:** Yunjin Li, Qiyue Xu, Lu Ma, Duojiao Wu, Jie Gao, Geng Chen, Hua Li

**Affiliations:** ^1^ Center for Bioinformatics and Computational Biology Shanghai Key Laboratory of Regulatory Biology Institute of Biomedical Sciences School of Life Sciences East China Normal University Shanghai China; ^2^ Institute of Clinical Science Zhongshan Hospital Fudan University Shanghai China; ^3^ Department of Cardiology Shanghai Institute of Cardiovascular Diseases Shanghai Xuhui District Central Hospital & Zhongshan‐xuhui Hospital Zhongshan Hospital Fudan University Shanghai China

**Keywords:** ACE2, COVID‐19, gene expression, SARS‐CoV‐2

## Abstract

Recent retrospective studies of severe acute respiratory syndrome coronavirus 2 (SARS‐CoV‐2) disease (COVID‐19) revealed that the patients with common comorbidities of cancers and chronic diseases face significantly poorer clinical outcomes than those without. Since the expression profile of ACE2, a crucial cell entry receptor for SARS‐CoV‐2, could indicate the susceptibility to SARS‐CoV‐2 infection, here we systematically dissected ACE2 expression using large‐scale multi‐omics data from 30 organs/tissues, 33 cancer types and some common chronic diseases involving >28 000 samples. It was found that sex and age could be correlated with the susceptibility of SARS‐CoV‐2 infection for certain tissues. Strikingly, ACE2 was up‐regulated in cervical squamous cell carcinoma and endocervical adenocarcinoma, colon adenocarcinoma, oesophageal carcinoma, kidney renal papillary cell carcinoma, lung adenocarcinoma and uterine corpus endometrial carcinoma compared to controls. Furthermore, the patients with common chronic diseases regarding angiocardiopathy, type 2 diabetes, liver, pneumonia and hypertension were also with higher ACE2 expression compared to related controls, which were validated using independent data sets. Collectively, our study may reveal a novel important mechanism that the patients with certain cancers and chronic diseases may express higher ACE2 expression compared to the individuals without diseases, which could lead to their higher susceptibility to multi‐organ injury of SARS‐CoV‐2 infection.

## 1 INTRODUCTION

As of 24 May 2020, the outbreak of severe acute respiratory syndrome coronavirus 2 (SARS‐CoV‐2) disease (COVID‐19) had resulted in >345 000 deaths among >5 400 000 infected cases worldwide. An increasing number of retrospective cohort studies regarding the patients with COVID‐19 throughout China revealed that the patients with common comorbidities of cancers and chronic diseases are with poorer clinical outcomes (eg admission to intensive care unit or death) than those without.^1^, ^2^, ^3^, ^4^, ^5^ Although immune deregulation could be one potential factor accounting for it, other causes and the underlying mechanism are poorly understood. It has been shown that the expression of cell receptor ACE2 could indicate the risk degree to SARS‐CoV‐2 infection,^6^, ^7^ thus dissecting ACE2 expression in diverse physiological and pathological conditions is crucial for identifying the susceptible population to special care and developing corresponding drugs and the treatment of SARS‐CoV‐2 infection. However, the expression profile of ACE2 in common comorbidities of SARS‐CoV‐2 infected patients like cancers and chronic diseases is still largely unknown.

Here, we systematically analysed ACE2 expression using large‐scale multi‐omics data from a variety of organs/tissues and cancer types, as well as the common chronic diseases of heart, liver, diabetes, pneumonia and hypertension involving a total of >28 000 samples. The underlying mechanisms of ACE2 expression variation in different conditions were also investigated. Moreover, we examined the association of sex and age with ACE2 expression in diverse tissues and cancers.

## 2 MATERIAL AND METHODS

### 2.1 Data collection and availability

We downloaded the pan‐cancer data of gene expression and DNA methylation (Methylation 450k) of The Cancer Genome Atlas (TCGA)^8^ from UCSC Xena. The gene expression matrices and related sample information of various human tissues were obtained from the Genotype‐Tissue Expression (GTEx).^9^ The batch effect removed and normalized data sets for TCGA and GTEx were also downloaded from a previous study.^10^ The gene expression data of different chronic diseases were obtained from Gene Expression Omnibus (GEO).^11^ Heart disease‐related data sets: GSE26887, GSE29819 and GSE59867, diabetes‐related datasets: GSE23343, GSE25724 and GSE77962. Chronic liver disease‐related data sets: GSE6764, GSE14323 and GSE89632. Pneumonia‐related data sets: GSE10667, GSE42830 and GSE110147. Hypertension‐related data sets: GSE53408 and GSE113439.

### 2.2 Statistics analysis

For testing the expression differences of ACE2 between two comparing groups, we employed Student's *t* test and defined *P* < 0.05 as significant. In order to examine the methylation level changes, we averaged the normalized beta values from TCGA pan‐cancer methylation data of the probes mapped to the promoter region (−1500 bp to −200bp upstream to the transcription start site [TSS]) of ACE2 to represent its methylation level. Then, Student's *t* test was employed to check the methylation differences, and *P* < 0.05 was considered as significant.

### 2.3 Construction of gene regulatory network

We inferred the gene regulatory network for different tissues of GTEx and each TCGA cancer using the SCENIC pipeline (version 1.1.0.1).^12^ First, the gene sets co‐expressed with TFs were identified using GENIE3.^13^ Then, the putative direct‐binding targets of TFs were identified by employing RcisTarget (version 1.2.1).^12^ Finally, the regulatory networks were identified with the SCENIC pipeline, and the regulons with less than 10 target genes were removed from downstream analysis.

## 3 RESULTS

### 3.1 ACE2 expression is associated with sex and age for certain tissues

**FIGURE 1:**
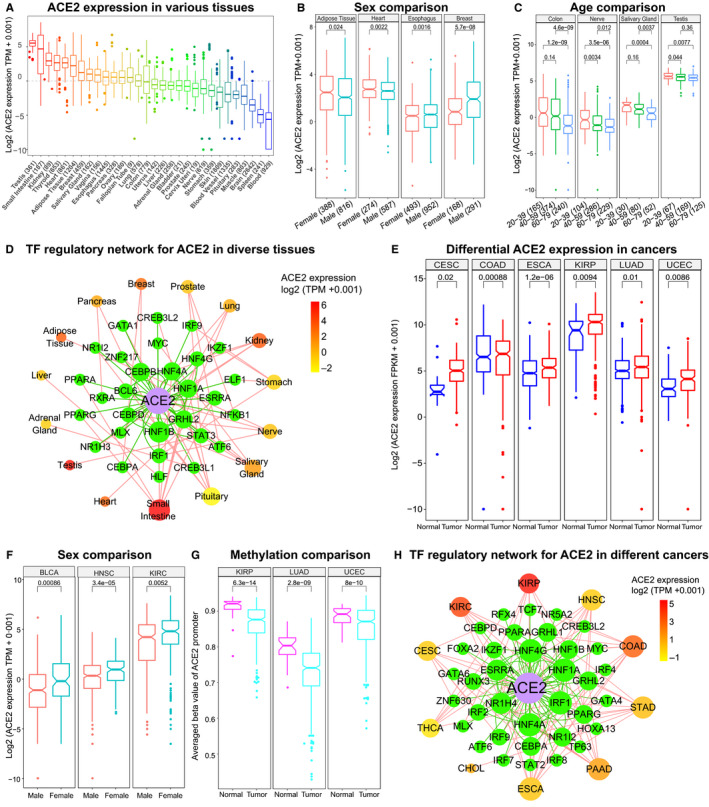
Expression and regulation of ACE2 in diverse normal tissues and cancers. A, Boxplot showing ACE2 expression in 30 different organs/tissues from GTEx project. B, ACE2 Expression differences between sexes in tissues. C, Boxplot displaying ACE2 expression comparison between distinct age groups. D, Transcription factor regulatory network for ACE2 in different tissues. Green nodes represent the detected transcription factors regulating ACE2. The intensity of the colour for tissues denotes the averaged expression level ACE2 in corresponding tissues. E, Boxplot displaying the up‐regulation of ACE2 in related cancers. F, Expression comparison between males and females in cancers. G, DNA methylation level comparison between males and females for ACE2 in tumours. H, Transcription factor regulatory network for ACE2 in cancers. Green nodes denote the detected transcription factors regulating ACE2. The intensity of the colour for cancers stands for the averaged expression level ACE2 in corresponding cancers. Note: Student's t test was used to check the significance. CESC, cervical squamous cell carcinoma and endocervical adenocarcinoma; COAD, colon adenocarcinoma; ESCA, oesophageal carcinoma; KIRP, kidney renal papillary cell carcinoma; LUAD, lung adenocarcinoma; UCEC, uterine corpus endometrial carcinoma; BLCA, bladder urothelial carcinoma; HNSC, head and neck squamous cell carcinoma; KIRC, kidney renal clear cell carcinoma; THCA, thyroid carcinoma; STAD, stomach adenocarcinoma; PAAD, pancreatic adenocarcinoma; CHOL, cholangiocarcinoma

We first profile ACE2 expression using the expression data of 30 organs/tissues containing >17 300 samples from GTEx^9^ and found that ACE2 was highly expressed in a set of tissues including testis, small intestine, kidney, thyroid, heart, adipose tissue and breast (Figure 1A), suggesting that these tissues may be vulnerable to SARS‐CoV‐2 infection, which is supported by recent clinical findings.^2^, ^14^ Interestingly, significant ACE2 expression differences between sexes were observed in adipose tissue, heart, breast and oesophagus (Figure 1B, *P* < 0.05). Moreover, differential ACE2 expression was detected between distinct age groups (20‐39, 40‐59 and 60‐79) of a set of tissues such as testis, salivary gland, colon, nerve, liver, adrenal gland and ovary (Figure 1C, *P* < 0.05). Thus, sex and age could be correlated with the susceptibility of SARS‐CoV‐2 infection for certain tissues. Gene regulatory network analysis of transcription factor (TF)‐ACE2 suggested that the dynamic changes of TF regulatory networks may account for the expression variation of ACE2 across tissues (Figure 1D).

### 3.2 ACE2 expression is up‐regulated in a set of cancers

We further explored ACE2 using multi‐omics data of 33 cancer types with >10 500 samples from TCGA.^8^ Remarkably, ACE2 was significantly up‐regulated in cervical squamous cell carcinoma and endocervical adenocarcinoma, colon adenocarcinoma, kidney renal papillary cell carcinoma, oesophageal carcinoma, lung adenocarcinoma and uterine corpus endometrial carcinoma compared to controls (Figure 1E, *P* < 0.05), suggesting that the patients with these tumours may face higher injury risk than the individuals without cancer after SARS‐CoV‐2 infection. The result could help explain the high risk of severe clinical outcomes for SARS‐CoV‐2 infected patients with cancers.^1^, ^2^, ^3^, ^4^, ^5^ Moreover, ACE2 showed significant expression differences between sexes in bladder urothelial carcinoma, head and neck squamous cell carcinoma, and kidney renal clear cell carcinoma (Figure 1F, *P* < 0.05). But no significant association between ACE2 expression and the age of cancer patients was observed. Moreover, further analysis suggested that methylation level changes of ACE2 promoter and the dynamics of TF‐ACE2 regulatory networks could be responsible for the expression difference of ACE2 between tumours and controls (Figure 1G,H, *P* < 0.05).

### 3.3 Patients with common chronic diseases show up‐regulated ACE2 expression

**FIGURE 2:**
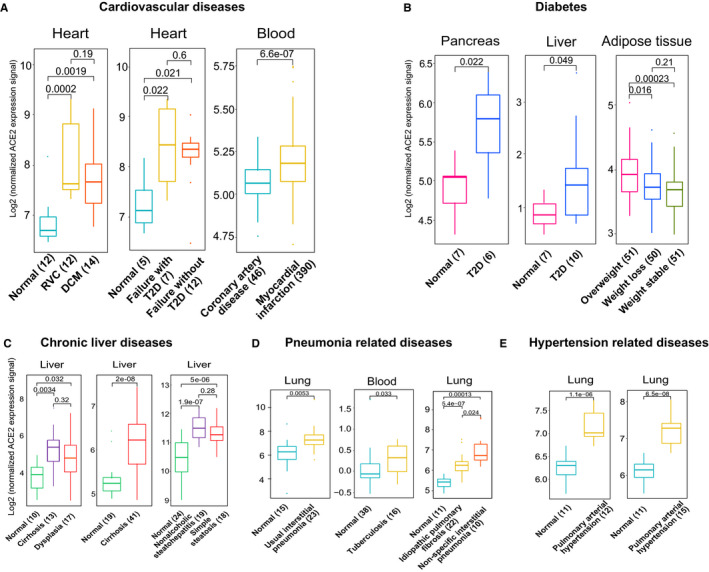
Expression profile of ACE2 in common chronic diseases. A, Expression comparison of ACE2 in cardiovascular diseases. Left (GSE29819): ACRV represents arrhythmogenic right ventricular cardiomyopathy; DCM denotes idiopathic dilated cardiomyopathy. Middle (GSE26887): Normal heart and heart failure with and without T2D. Right (GSE59867). B, Boxplot showing expression comparison of ACE2 in diabetes‐related data sets. Left (GSE25724); middle (GSE23343); right (GSE77962): abdominal subcutaneous white adipose tissue of overweight, weight loss and weight stable period. C, Expression comparison of ACE2 in chronic liver diseases. Left (GSE6764); middle (GSE14323); right (GSE89632). D, Expression comparison of ACE2 in pneumonia‐related diseases. Left (GSE10667); middle (GSE42830); right (GSE110147). E. Boxplot displaying expression comparison of ACE2 in hypertension‐related diseases. Left (GSE53408); right (GSE113439). Note: Student's t test was employed to examine the significance

Since the SARS‐CoV‐2 infected patients with certain common chronic diseases also face a relatively high risk of poor clinical outcomes,^2^, ^3^, ^4^, ^5^ we further explored the underlying mechanism by profiling ACE2 expression with the expression data of common chronic diseases regarding angiocardiopathy, type 2 diabetes (T2D), liver, hypertension and pneumonia obtained from GEO.^11^ Surprisingly, individuals with cardiovascular diseases could express higher ACE2 expression than those without heart diseases (non‐failing hearts) using three independent data sets (Figure 2A, *P* < 0.01). Moreover, ACE2 was up‐regulated in patients with T2D compared to the individuals without T2D in three independent datasets (Figure 2B, *P* < 0.05). The liver tissues with chronic diseases such as cirrhosis, dysplasia, non‐alcoholic steatohepatitis, and simple steatosis also could express higher levels of ACE2 than that of normal liver tissues with three independent data sets (Figure 2C, *P* < 0.05). Furthermore, higher expression of ACE2 was detected in pneumonia compared to normal controls using three independent data sets (Figure 2D, *P* < 0.05). Additionally, the up‐regulation of ACE2 was detected in patients with pulmonary arterial hypertension compared to healthy individuals in two independent data sets (Figure 2E, *P* < 0.01). Therefore, expression of ACE2 could be up‐regulated in patients with these common chronic diseases, which may result in greater susceptibility to the injury of SARS‐CoV‐2 infection.

## 4 DISCUSSION

In summary, our findings may reveal a novel important mechanism that the patients with certain cancers and chronic diseases could express higher ACE2 expression compared to the individuals without diseases, which may lead to their higher susceptibility to multi‐organ injury of SARS‐CoV‐2 infection. We believe this study will largely facilitate better understanding and interpretation of the injury risk and clinical outcomes of SARS‐CoV‐2 infected patients with common comorbidities. Our results could be very practical for effective population protection and scientific clinical treatment.

## CONFLICT OF INTEREST

The authors confirm that there are no conflicts of interest.

## AUTHOR CONTRIBUTION

**Yunjin Li:** Data curation (equal); Formal analysis (equal); Investigation (equal); Methodology (equal); Writing‐review & editing (equal). **Qiyue Xu:** Data curation (equal); Formal analysis (equal); Investigation (equal); Methodology (equal); Writing‐review & editing (equal). **Lu Ma:** Data curation (equal); Formal analysis (equal); Investigation (equal); Methodology (equal); Writing‐review & editing (equal). **Duojiao Wu:** Investigation (equal); Writing‐review & editing (equal). **Jie Gao:** Investigation (equal); Writing‐review & editing (equal). **Geng Chen:** Conceptualization (equal); Data curation (equal); Funding acquisition (equal); Investigation (equal); Project administration (equal); Resources (equal); Supervision (equal); Writing‐original draft (equal); Writing‐review & editing (equal). **Hua Li:** Conceptualization (equal); Data curation (equal); Funding acquisition (equal); Investigation (equal); Project administration (equal); Resources (equal); Supervision (equal); Writing‐original draft (equal); Writing‐review & editing (equal).

## Data Availability

All the data used in this study were downloaded from public databases as stated in the manuscript.
